# Assembling Native Elementary Cellulose Nanofibrils via a Reversible and Regioselective Surface Functionalization

**DOI:** 10.1021/jacs.1c06502

**Published:** 2021-10-07

**Authors:** Marco Beaumont, Blaise L. Tardy, Guillermo Reyes, Tetyana V. Koso, Elisabeth Schaubmayr, Paul Jusner, Alistair W. T. King, Raymond R. Dagastine, Antje Potthast, Orlando J. Rojas, Thomas Rosenau

**Affiliations:** † Department of Chemistry, Institute of Chemistry of Renewable Resources, University of Natural Resources and Life Sciences, Vienna, Konrad-Lorenz-Str. 24, 3430 Tulln, Austria; ‡ Department of Bioproducts and Biosystems, School of Chemical Engineering, Aalto University, P.O. Box 16300, Espoo FI-00076, Finland; § Materials Chemistry Division, Department of Chemistry, University of Helsinki, AI Virtasen aukio 1, FI-00560 Helsinki, Finland; ∥ Department of Chemical & Biomolecular Engineering, The University of Melbourne, Grattan Street, Parkville, Victoria 3010, Australia; ⊥ Bioproducts Institute, Department of Chemical & Biological Engineering, Department of Chemistry and Department of Wood Science, The University of British Columbia, Vancouver, BC V6T 1Z3, Canada; ¶ Johan Gadolin Process Chemistry Centre, Åbo Akademi University, Porthansgatan 3, Åbo/Turku FI-20500, Finland

## Abstract

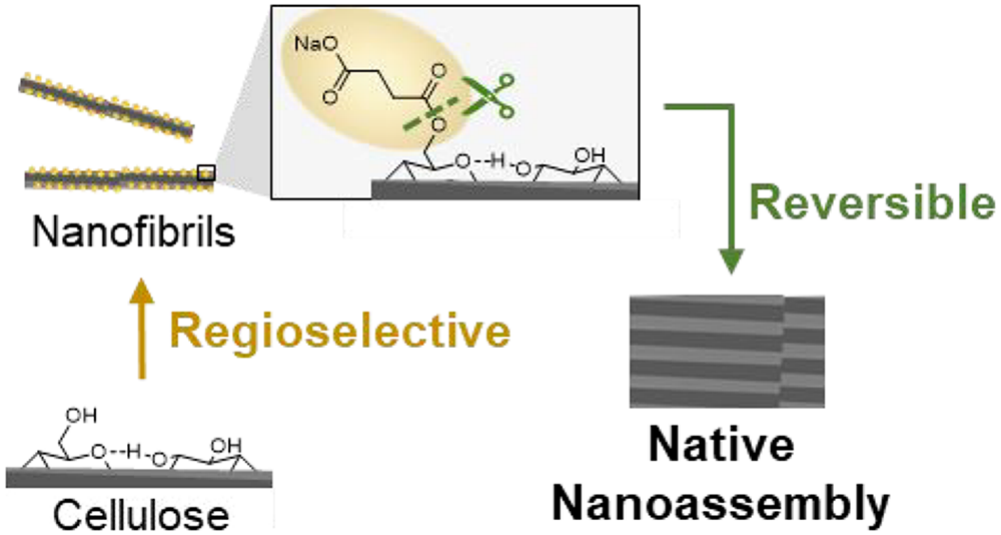

Selective surface modification of biobased fibers affords effective individualization and functionalization into nanomaterials, as exemplified by the TEMPO-mediated oxidation. However, such a route leads to changes of the native surface chemistry, affecting interparticle interactions and limiting the development of potential supermaterials. Here we introduce a methodology to extract elementary cellulose fibrils by treatment of biomass with *N*-succinylimidazole, achieving regioselective surface modification of C6-OH, which can be reverted using mild post-treatments. No polymer degradation, cross-linking, nor changes in crystallinity occur under the mild processing conditions, yielding cellulose nanofibrils bearing carboxyl moieties, which can be removed by saponification. The latter offers a significant opportunity in the reconstitution of the chemical and structural interfaces associated with the native states. Consequently, 3D structuring of native elementary cellulose nanofibrils is made possible with the same supramolecular features as the biosynthesized fibers, which is required to unlock the full potential of cellulose as a sustainable building block.

## 1 Introduction

New bio-based feedstock streams are needed to develop sustainable materials that surpass in performance the prevalent synthetic counterparts. In this regard, the isolation from biomass of native structural components of high intrinsic cohesion and defined morphology presents a unique opportunity.^1^ The biogenesis of cellulose chains from synthase systems results in polymeric constructs with one of the highest strengths reported to date. Driven by supramolecular interactions, tightly packed elementary fibrils are formed (diameter of approximately 3–4 nm) exhibiting a remarkable tensile strength and modulus, reaching values as high as 7 GPa^2,3^ and 140 GPa,^4−6^ respectively. They can be readily obtained from forestry, ocean, and agricultural side-streams, and their promise in high-performance sustainable materials has triggered great interest over the past decade.^1,3^ However, there is a standing need for new, green routes to re-engineer the native cellulosic supramolecular interactions into macroscale materials, ideally in line with green chemistry and technology principles.

Mechanical fibrillation of the plant cell wall, following optional pretreatments (*e.g*., enzymatic), results in bundles of cellulose nanofibrils (CNFs) carrying residual hemicelluloses, which dominate most supramolecular interactions, given their higher surface activity and reactivity.^7,8^ The most prominent chemical pretreatment that enables individualization into elementary fibrils is a nearly regioselective modification by oxidation of the cellulose’s primary OH groups, namely, TEMPO-mediated oxidation (2,2,6,6-tetramethylpiperidin-1-yl-oxyl being the oxidant).^9^ An alternative approach is the periodate oxidation and subsequent Pinnick oxidation,^10^ which converts the secondary alcohol groups of cellulose into carboxyl moieties after C2–C3 bond cleavage. While these modifications are commonly used, they also have major drawbacks, including those related to chemical degradation and their irreversibility. For instance, complete surface modification of the nanofibrils by TEMPO-oxidation has been demonstrated to occur in alkaline media,^11,12^ causing a drastic decrease in molar mass,^13^ which even occurs at moderate oxidation conditions (in the range of 0.5 mmol COOH g^–1^ of CNFs).^14,15^ These effects limit the mechanical performance and the corresponding prospects of the assembled materials.^16^ In addition, TEMPO-oxidation is irreversible, yielding nanofibrils with carboxylated surfaces, which prevent the strong interactions that are otherwise present in native supramolecular structures.^11^ By contrast, cellulose esters can be cleaved by saponification, enabling the recovery of the intrinsic surface functionality and cohesive interactions.^17^ This latter observation inspired our study, as presented in this discussion.

**Figure 1:**
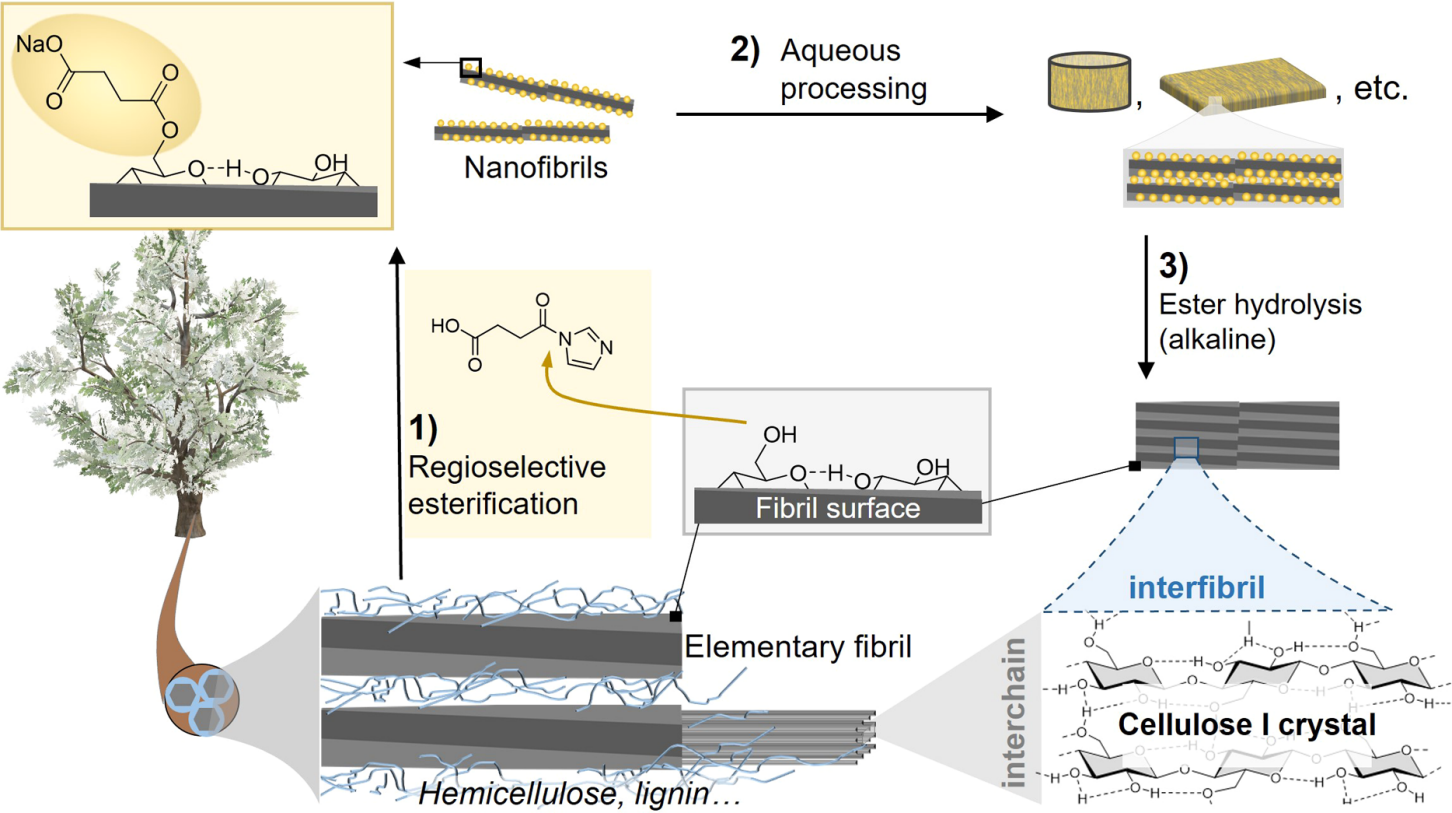
Elementary succinylated cellulose nanofibrils (C6SA-CNF) were produced from renewable cellulose fibers by regioselective esterification of the primary C6-OH through reaction with *N*-succinyl imidazole followed by fibrillation in a high-pressure homogenizer (1). As is the case of conventional CNF, C6SA-CNF forms hydrogels of given shapes (2) but is amenable to removal of the installed groups by facile hydrolysis. Hence, 3D structures can be formed, which are composed of the elementary fibrils of cellulose in its type I, the native cellulose allomorph (3). The hydrolysis treatment induces strong interfibrillar interactions, rivaling those present in the native cellulose I crystals.

Herein, cellulose fibers were deconstructed into elementary fibrils by regioselective modification with *N-*succinylimidazole (Figure 1). This surface modification is introduced on the entire fibril surface, with very high selectivity toward the primary hydroxyl group of cellulose.^18−20^ The method is mild and does not lead to dissolution nor influence the inherent physicochemical properties, such as crystallinity and molar mass, while preserving the morphology of the elementary structures, even at complete surface functionalization. Preserving such native properties is essential to maintain the excellent mechanical properties of cellulose.^4,5^ The negative carboxylate charge facilitates individualization into elementary nanofibrils, which can be processed, in a fashion similar to TEMPO-CNF, into an arbitrary shape, e.g., by extrusion, wet-spinning, or film formation.^1,21,22^ While for TEMPO-CNF the interfacial interactions are permanently impacted by carboxyls groups, the succinyl ester moieties can be hydrolyzed (saponified) under mild basic conditions to recover the pristine chemical structure of cellulose I, re-establishing its native supramolecular interactions. This offers a unique route to enhance processability of plant biomass into materials while recovering their performance potential. We describe the efficiency of the reaction, its regioselectivity, and reversibility by nuclear magnetic resonance, infrared spectroscopy, and gel permeation chromatography. The morphology of the elementary fibrils is then evaluated using atomic force microscopy and scanning electron microscopy, revealing the fibril morphology and size, which match those of the native fibrils. Finally, we demonstrate processing in aqueous media that leads to hydrogels, aerogels, and films, the latter being compared for their mechanical properties before and after saponification. The presented new avenue to engineer cellulosic building blocks will unlock new opportunities in the fabrication of sustainable, high-strength, and lightweight materials.

## 2 Results and Discussion

**Figure 2:**
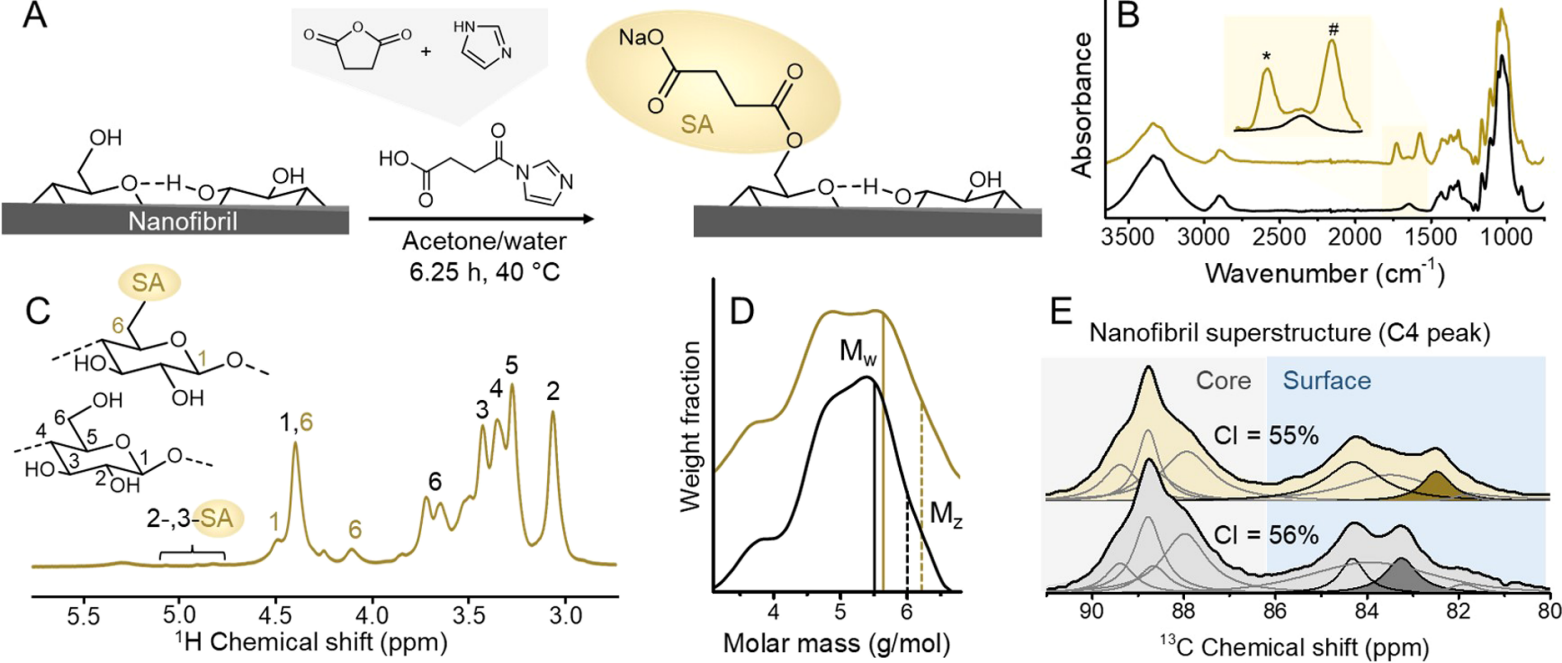
*N*-Succinyl imidazole, the acyl transfer agent, was produced in situ through reaction of imidazole and succinic anhydride (A). The wet cellulose fibers were treated at 40 °C for 6.25 h in an acetone/water mixture to introduce the succinyl group onto the primary C6-OH of cellulose (92% selectivity). The IR spectrum clearly demonstrates the successful introduction of the succinyl group (*λ = 1723 cm^–1^, ^#^λ = 1568 cm^–1^) (B), and the regioselectivity was studied by solution-state nuclear magnetic resonance, showing only minor modification of the C2- and C3-OH (2-,3-SA) (C). The molar mass (both weight- and *z*-averaged) increased through introduction of the succinyl group (D). The superstructure and crystallinity index (CI), studied by solid-state NMR, were preserved, confirming that the reaction was confined to the accessible (amorphous) nanofibril surface (E).

The preparation of structural assemblies based on pristine, individualized, cellulose nanofibrils is shown schematically in Figure 1. The native cellulose fibers were regioselectively modified by succinylation of the surface C6-OH glucose repeating units. Thereby, CNFs with the same selectivity (C6) and largely similar chemical functionality (succinate vs carboxylate) as the well-known TEMPO-CNF were obtained. The process was mediated via a reactive acylimidazole intermediate^18−20^ and can be applied directly to never-dried biomass, i.e., wet pulp fibers, commonly used for the preparation of CNFs.^10,12,18^ The reaction is water-promoted and even proceeds faster in the presence of water as it is an acyl transfer rather than a classical esterification.^19,20^ Never-dried dissolving-grade beech pulp was used as starting material. The modification was conducted in an acetone/water solvent system in the presence of succinic anhydride (1.0 molar equiv based on cellulose monomer unit) and imidazole (1.5 equiv) that generate the acylating agent *N*-succinylimidazole in situ (Figure 2A). The esterification was completed after 6.25 h and the final carboxylate content can be tailored by varying the reaction conditions (Figure S1). Afterward, acetone and the remaining reactants were removed by thorough washing with water. Successful introduction of the succinyl group was demonstrated by infrared spectroscopy, which confirmed the presence of the carbonyl bands at 1568 cm^–1^ and 1723 cm^–1^ (Figure 2B). The modified cellulose was directly compared with the starting material, never-dried cellulose fibers, which is referred to as “reference”. The degree of substitution, determined by conductometric titration (Figure S2) was 0.25 ± 0.02, corresponding to a carboxylate content of 1.3 ± 0.1 mmol/g (Table S1). These values were also confirmed by diffusion-edited liquid-state NMR in the [P_4444_][OAc]:DMSO-*d*_6_ solvent.^23,24^ This analysis also afforded information on the regioselectivity of the modification (Figure 2C),^19,20^ and NMR peaks were assigned through the respective multiplicity-edited heteronuclear single-quantum correlation (HSQC) spectrum (Figure S3). The calculated total DS and that specific to the primary C6-OH were 0.24 and 0.22, respectively, yielding a reaction regioselectivity of 92%. Based on the crystallite size, we calculated the theoretically available C6-OH number of 0.22 (Table S1) and concluded that the entire number of available C6-OH groups at the surface of the elementary fibrils had reacted, a regioselectivity that was previously only possible with TEMPO-oxidation.^11^ Solid-state NMR (Figure S4 and Figure 2E), was used to gather information on the fibril superstructure based on deconvolution of the C4 peak, composed of a chemically nonaccessible, crystalline core and an accessible surface.^25,26^ One of the peaks corresponding to the chemically accessible surface—shaded in dark gray and bronze for reference and C6SA-CNF, respectively—is clearly shifted upfield (from 83.2 to 82.5 ppm) upon surface modification. We assign this peak to C4 of the C6-succinylated glucopyranose surface units and further conclude a homogeneous surface modification. In addition, it is clearly shown that the fibril superstructure and crystallinity (Table S1) is well preserved, which contrasts with NMR results of TEMPO-oxidized CNF (Figure S5). Compared to oxidative treatments, such as periodate or TEMPO-oxidation, the molar mass is not reduced (although changes occurred due to the introduction of succinyl groups, Figure 2D). The increase of the weight-averaged degree of polymerization upon modification is the result of removal of hemicelluloses and/or low molar mass cellulose fractions; the former reason, in fact, was confirmed by solid-state NMR measurements (Table S1).

The effect of the fibrillation degree on the rheological properties was assessed for C6SA-CNF and compared to that of TO-CNF (prepared under neutral conditions)^14^ (Figure S6), showing that C6SA-CNF underwent a slightly more extensive fibrillation. Moreover, we show that, similar to other CNF types, C6SA-CNF exhibits strong shear-thinning and the rheological properties typical of a gel, due to its dominant elastic behavior (*G*′ > *G*″) (Figure S7).

**Figure 3:**
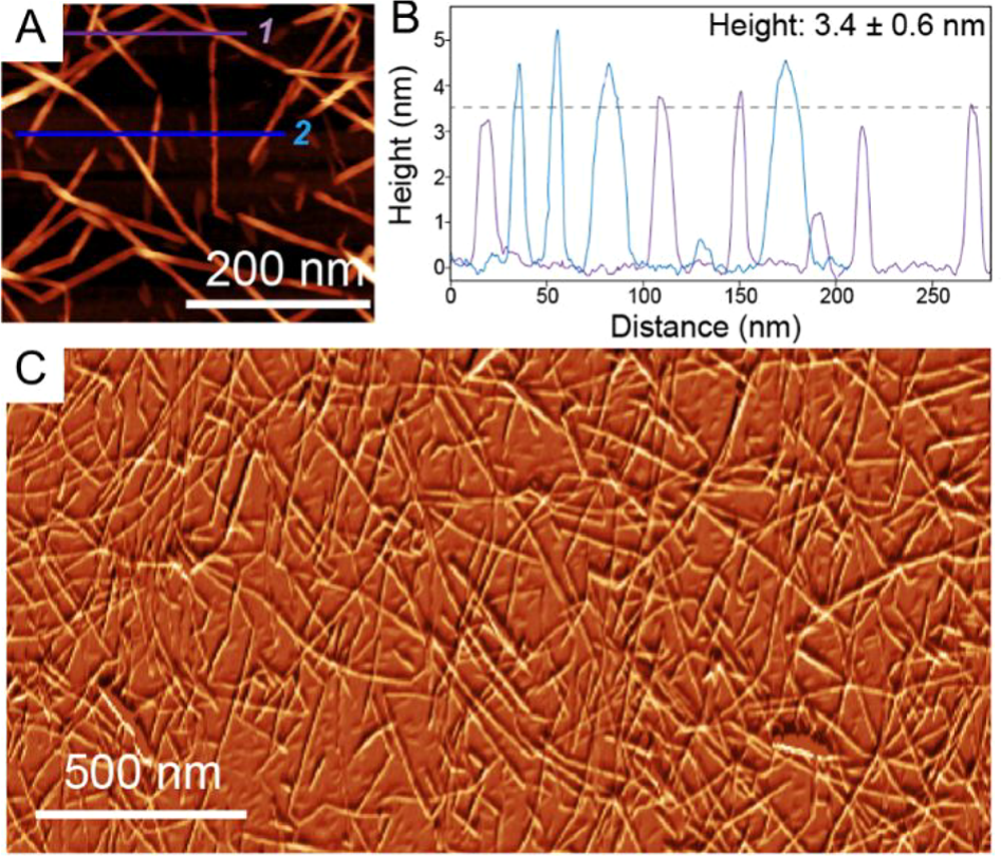
Atomic force microscopy imaging of elementary fibrils of C6SA-CNF (A) and height profiles obtained for 9 fibrils (B). The overview microscopy image indicates well-dispersed and homogeneous fibrils (C).

The morphology and dimensions of the nanofibrils were evaluated using atomic force microscopy (Figure 3). In the given scanning areas, the fibrils appeared rather homogeneous (Figure 3A); the absence of bundling suggested complete individualization into elementary nanofibrils through our process. Figure 3A includes over 50 fibrils that were clearly individualized, and the corresponding height profiles revealed rather uniform height values across the sample (Figure 3A, respective phase and amplitude images in Figure S9), e*.*g*.,* a narrow height distribution (3.4 ± 0.6 nm, Figure S10). These values agree with those measured for elementary fibrils (also referred to as crystallite size) of approximately 4 nm (Table S1). This clearly indicates a complete fibrillation of the cellulose fiber into its elementary nanofibrils. Interestingly, a small fraction of the deposits on the surface were smaller fibrils with heights below 1 nm and lengths of ca. 20 nm. These may be cellulosic fragments resulting from the pulping process, fibrillation treatment, and/or residual hemicelluloses.

**Figure 4:**
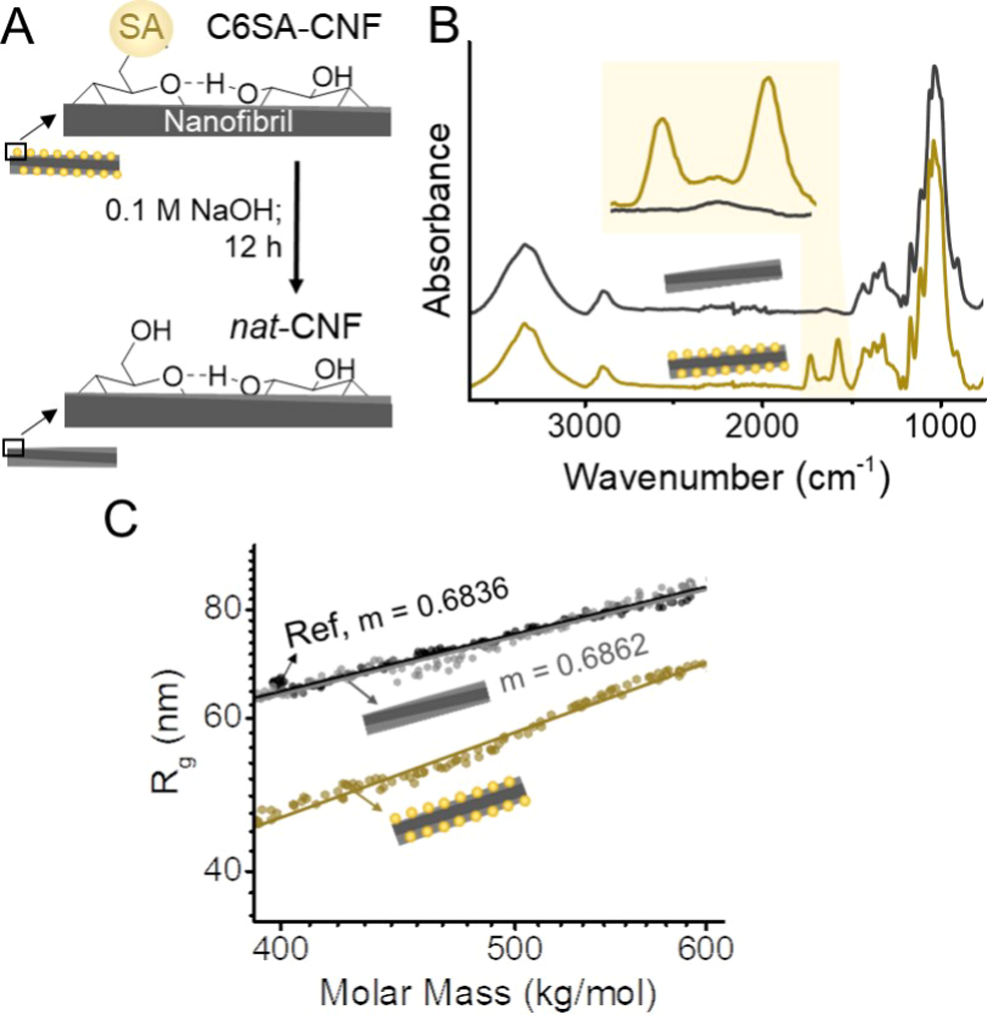
Regioselective introduction of succinyl groups in C6SA-CNF (top-left) can be reversed by alkaline treatment (0.1 M NaOH, bottom-left) (A), as shown by IR spectroscopy, through the disappearance of the carbonyl bands (highlighted in light yellow) (B), as well as by the conformation plot from light scattering analysis (C). The conformation plot (molar mass vs radius of gyration, *R*_g_) of the NaOH-treated C6SA-cellulose and its slope (*m*) is almost identical compared to the native sample (assigned as ref in C), confirming the reversible nature of the succinylation (lines in C are linear fittings of the respective conformation plot).

The chemistry used herein is nondestructive, which contrasts significantly with the oxidative routes conventionally used for modification or enhanced dispersion of nanocelluloses. Moreover, the introduced succinate ester is stable under conventional conditions (pH range 3–9). This enables a wide and versatile application range for C6SA-CNF. It is well known that ester groups are susceptible to hydrolysis, i.e., saponification. This is applicable to our modified CNF, *i.e*., by treatment with 0.1 M NaOH, yielding native elementary CNF, which we submit to be similar to the natural form (herein referred to as *nat*-CNF) (Figure 4A). It can be reasonably proposed that pristine cellulose is restored after hydrolysis, upon removal of the succinate groups, as demonstrated by IR spectroscopy (Figure 4B) through the disappearance of the carbonyl bands and as also shown by gel permeation chromatography with multiangle light scattering detection. Both weight- and *z*-average molar mass of the succinylated cellulose were clearly reduced upon saponification by loss of the succinate, returning to values similar to those of the reference sample, which is also well reflected in the respective molar mass distributions (Table S1 and Figure S8). Moreover, the conformation plot of *nat*-cellulose showed that after hydrolytic treatment the shape and dependency of the radius of gyration and molar mass of the dissolved cellulose sample returned to the initial, reference state (Figure 4C, black and gray fits). Generally, the alkaline treatment induced a cross-linking/gelation of the C6SA-CNF, similar to the behavior of carboxylated CNF under acidic conditions (protonation) or in the presence of multivalent ions (ionic cross-linking through replacement of Na^+^ counterions). The alkaline treatment can be conducted directly from C6SA-CNF in sodium form or from C6SA-CNF hydrogels prepared by ionic cross-linking.

The effect of various treatments on the properties of prepared nanopapers was evaluated through the increase in thickness in relation to the dry film upon swelling in water (Figure S12). In all cases, treatments were conducted on nanopapers; base- and acid-treated samples were subsequently washed with water to remove soluble residues. Without base or acid pretreatment, both C6SA-CNF and TO-CNF films swelled significantly, as shown by the 8.5- and 15-fold increase in thickness, respectively. The lower increase of C6SA-CNF is most probably related to the fact that the succinyl groups of C6SA-CNF are less hydrophilic compared to the carboxylate groups in TO-CNF. In contrast, the thickness increased significantly less after mild acid treatment (0.01 M HCl), due to the protonation of the carboxylate groups and thereby induced gelation (due to lower electrostatic repulsion). In comparison, upon exposure to a base treatment (0.1 M NaOH), the thickness of TO-CNF films increased to a similar level as that in pure water (13-fold), while C6SA-CNF swelling was severely limited (3-fold), which is a result of the ester hydrolysis into *nat*-CNFs and the induced gelation. The water-swollen nanopapers are hereafter referred to as hydrogels.

**Figure 5:**
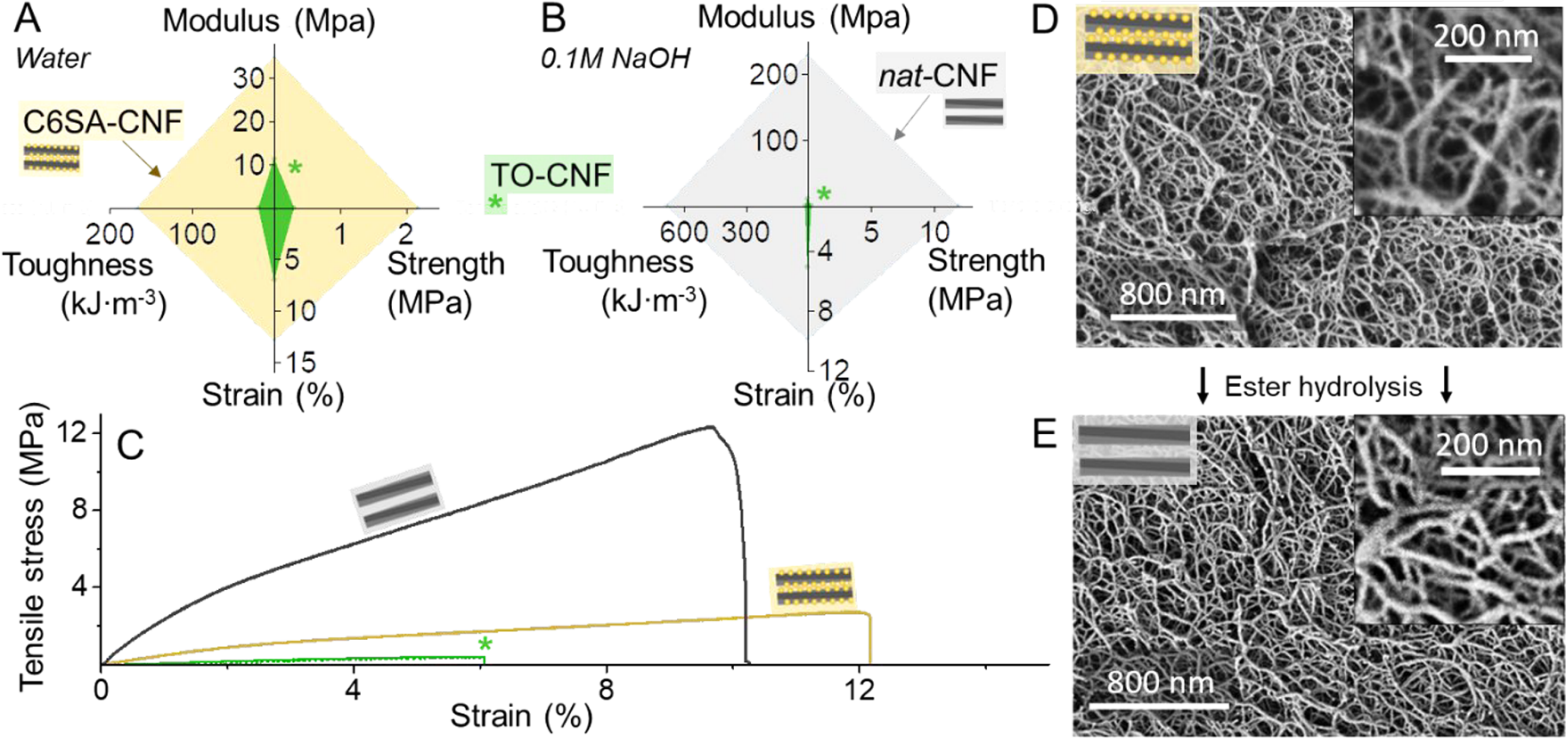
Mechanical performance of hydrogels produced from C6SA-CNF and TO-CNF, which were equilibrated in water prior to (A) and after treatment in 0.1 M NaOH (B). C6SA-CNF exhibits a stronger mechanical stability in water compared to TO-CNF, which was especially pronounced after NaOH treatment, causing ester hydrolysis to yield *nat*-CNF. Representative tensile curves of the systems compared in A and B (C). Networks of C6SA-CNF (D) and *nat*-CNF (E) in an aerogel. The schematic figures illustrate the possible nanofiber surface structure with and without prior NaOH treatment.

The mechanical properties of the hydrogels, always measured after equilibration in deionized water regardless of pretreatment, were evaluated by tensile testing, Figure 5A,B, with representative tensile strain curves shown in Figure 5C. As expected, both TO-CNF and C6SA-CNF hydrogels showed a rather low mechanical strength when immersed in water, 0.3 and 2.2 MPa, respectively. The higher strength of C6SA-CNF hydrogels results from the lower swelling tendency of C6SA-CNF, which helps to resist the effect of water in the wet conditions. C6SA-CNF and TO-CNF hydrogels presented similar tensile strengths under acidic conditions, 12.5 and 20 MPa, respectively (Figure S13); the elastic modulus and toughness followed a similar ranking. Upon 0.1 M NaOH treatment, the mechanical properties of the TO-CNF hydrogel were similar to those measured in water. In contrast, the tensile strength of NaOH-treated C6SA-CNF (*nat*-CNF, Figure 5B) was of the same order as that of C6SA-CNF, 12 MPa, when the aqueous medium was changed to an acid medium (Figure S13). This demonstrates that the ester hydrolysis (saponification) induced physical cross-linking, due to the removal of the charged ester group, restoring the hydrogen-bond network of native celluloses.

The hydrogels were solvent-exchanged with acetone and supercritically dried to yield nanofibrillar aerogels. The prepared C6SA-CNF aerogel featured a specific surface area of 415 m^2^ g^–1^, which is comparable to the one of a prepared TEMPO-oxidized CNF aerogel (see Figure S11 for further information). Scanning electron microscopy analysis showed that the fibrillar network structures were very similar prior to and after removal of the succinyl groups (Figure 5D,E). We speculate that the network formed after saponification resembles that of native cellulose nanofibrils (Figure 5).

**Figure 6:**
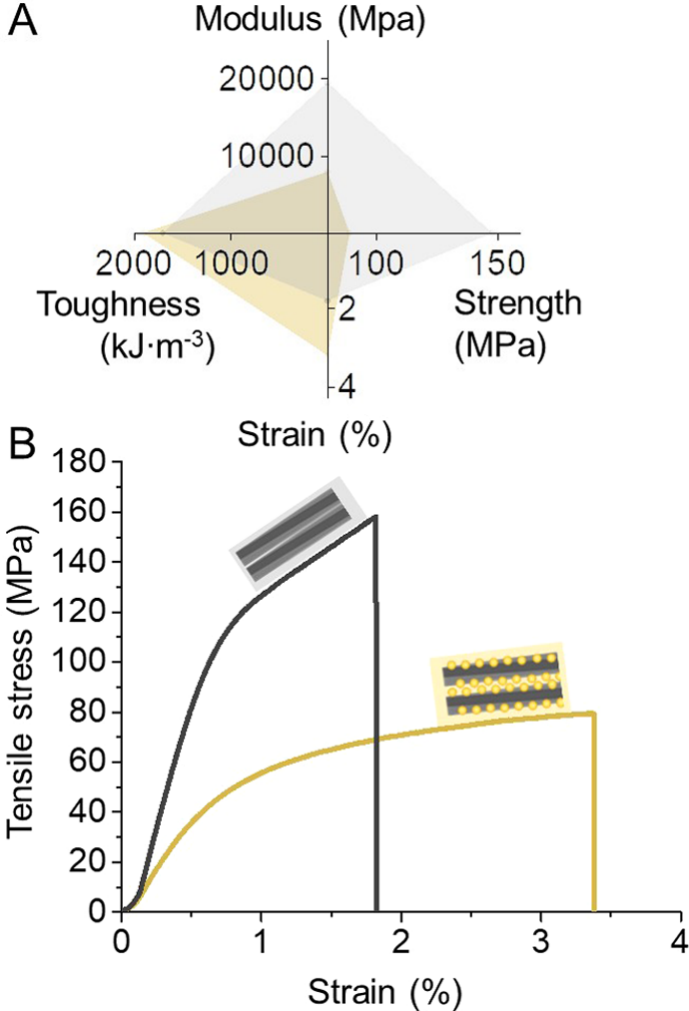
Mechanical properties of dried nanopapers of C6SA-CNF (yellow profile and shading) and *nat*-CNF (gray profile and shading) (A). Representative stress–strain curves for the samples indicate the recovery of the native, interfibrillar cohesion of *nat*-CNF (B). The schematic figures in B illustrate the possible nanofibers surface structure of C6SA-CNF and *nat*-CNF.

As shown in Figure 5C, the supramolecular interactions in water, upon recovery of the native cellulose interfaces, resulted in an improved tensile strength, by ca. 6-fold, with a relatively small reduction of the strain at break, from 13% to 10%. By favoring supramolecular interactions, upon drying of *nat-*CNF hydrogels, the strain at break was on the other hand reduced, from 3.2% to 1.8% (Figure 6). The mechanical performance of the respective dry films is compared in Figure 6. A significant increase in strength (2.5-fold, from 79 MPa to 194 MPa) was realized. Likewise, the Young’s moduli increased from 7.9 GPa to 19.4 GPa. This suggests a significant improvement in the interfacial cohesion resulting from the recovery of the native supramolecular interactions after saponification.

The here-prepared *nat*-CNF showed a Young’s modulus (19 GPa) significantly higher than the moduli of nanopapers prepared from TEMPO-oxidized CNFs (≤10 GPa)^27−29^ or from mechanically fibrillated cellulose nanofibers (≤13.4 GPa).^30,31^ The tensile strength of *nat*-CNF, in comparison to reference materials, is moderately high.^27,30^ Considering the relatively low apparent density, 1.31 g cm^3^ (relative porosity of 16%) (Table S3), the specific tensile strength of *nat*-CNF is in the range of those of high-strength nanopapers. This suggests that the restored cellulose–cellulose interactions induce stronger cohesive forces, although they were not specifically optimized in the material formed herein. We expect that the mechanical performance can be further enhanced by densification of the nanopaper or with improved long-range order, i.e., with increased coherence between the fibrils.

## 3 Conclusions

In conclusion, we have shown that the presented regioselective succinylation approach enables complete isolation of elementary cellulose fibrils from wood pulp fibers, without compromising their crystalline structure and degree of polymerization. This contrasts with nanocellulose preparation methods based on oxidative treatments, such as the TEMPO-mediated and periodate oxidation. It is noteworthy that these chemical approaches are quite distinct, so that their efficiency may differ when applied to different biomasses, e.g., never-dried vs dried pulps. Moreover, we showed that the proposed modification enabled complete surface esterification of the available primary hydroxyl groups of the elementary fibril, i.e., full surface coverage, and thereby fibrillation into elementary nanofibrils. The resulting nanofibril dispersions could be processed using conventional approaches while allowing recovery of the native supramolecular interactions by a subsequent mild hydrolysis (saponification), which removed the succinate groups. The reversibility of the proposed functionalization enables structures composed of elementary nanofibrils exhibiting their native characteristics, which can help overcome the current limitations in mechanical performance of nanocelluloses.

Other implications can be foreseen from these “native” nanofibrils, such as optimized interactions with other plant-based polymers, e*.*g., hemicelluloses. Structures composed of native elementary cellulose fibrils, made available by our approaches, are expected to advance the developments and implementation of materials from sustainable building blocks. We expect that the introduced methods will push the upper strength boundaries of nanocelluloses and that their implementation will result in a wider range of high-end materials.

## 4 Experimental Section

### Materials

Cellulose fibers of high purity were provided as never-dried bleached beech sulfite dissolving pulp (50 wt % solid content) by Lenzing AG (Lenzing, Austria) and used in the production of the modified pulp sample (C6SA-cellulose). All chemicals were purchased from Sigma-Aldrich (Merck Life Science OY, Finland) at a minimum purity of 99% and were used as received.

### Preparation of Regioselectively Succinylated Cellulose Nanofibrils (C6SA-CNFs)

Never-dried cellulose fibers (20.0 g wet mass, 10 g dry mass, 61.7 mmol, 50 wt % solid content) was transferred into a flask. In a separate container, 30.8 mL of a 3 M solution of imidazole (6.30 g, 92.5 mmol, 1.5 molar equiv) in acetone was stirred with 61.7 mL of a 1 M succinic anhydride solution (6.17 g, 61.7 mmol, 1.0 molar equiv) in acetone for 10 min. Afterward this mixture was added to the cellulose fibers and mixed by stirring with a glass rod for 1 min. The container was closed and heated in an oven at 40 °C for 6.25 h. The reaction was stopped through addition of a saturated aqeuous solution of NaHCO_3_ and 30 min equilibration. To remove the unreacted SA and the imidazole from the cellulose, the pulp was washed by filtration with deionized water. The cellulose fibers were suspended in deionized water at 0.25 wt % solid content with a blender. The C6SA-cellulose suspension was fibrillated in a high-pressure homogenizer, Gaulin APV-1000 from AxFlow GesmbH (Premstätten, Austria). The homogenization of the fibers was done in five passes at a pressure of approximately 800 bar to yield a highly viscous and transparent dispersion of C6SA-CNF, which was stored at 8 °C. Further experimental details are given in the Supporting Information.

## Supporting Information Available

Physical properties before and after functionalization, optimization of the reaction conditions and further experimental details, materials characterization with respect to surface chemistry, molar mass, rheology, and surface structure; experimental procedure to prepare C6SA-CNF hydrogels, aerogels, and films, swelling behavior, porosity, and mechanical properties of dry films and hydrogels (PDF)
