# Supplementary material for: Assembling Native Elementary Cellulose Nanofibrils via a Reversible and Regioselective Surface Functionalization
Source: J Am Chem Soc. 2021 Oct 7;143(41):17040–6. doi: 10.1021/jacs.1c06502 (PMC8532154; doi:10.1021/jacs.1c06502)
Supplement: Supplementary file 1 — ja1c06502_si_001.pdf [file ja1c06502_si_001.pdf]

Supporting Information

Assembling Native Elementary Cellulose Nanofibrils via a Reversible and Regioselective Surface Functionalization

Marco Beaumont,[1#] Blaise L. Tardy,[2#] Guillermo Reyes,[2] Tetyana V. Koso,[3] Elisabeth Schaubmayr,[1] Paul Jusner, [1] Alistair W. T. King,[3] Raymond R. Dagastine,[4] Antje Potthast,[1] Orlando J. Rojas,[2,5]* and Thomas Rosenau[1,6]**

[1] Department of Chemistry, Institute of Chemistry for Renewable Resources, University of Natural Resources and Life Sciences Vienna (BOKU), Konrad-Lorenz-Straße 24, A-3430 Tulln, Austria.

[2] Department of Bioproducts and Biosystems, School of Chemical Engineering, Aalto University, P.O. Box 16300, Espoo FI-00076, Finland.

[3] Materials Chemistry Division, Department of Chemistry, University of Helsinki, AI Virtasen aukio 1, FI-00560 Helsinki, Finland.

[4] Department of Chemical & Biomolecular Engineering; The University of Melbourne, Grattan Street, Parkville, Victoria, 3010, Australia.

[5] Bioproducts Institute, Department of Chemical & Biological Engineering, Department of Department of Chemistry and Wood Science, The University of British Columbia, Vancouver, BC V6T 1Z3, Canada.

[6]Johan Gadolin Process Chemistry Centre, Åbo Akademi University, Porthansgatan 3, Åbo/Turku FI-20500, Finland.

[#]These authors contributed equally to this work. *Corresponding authors: thomas.rosenau@boku.ac.at, orlando.rojas@ubc.ca, marcobeaumont1@gmail.com

KEYWORDS: Transesterification, functional nanoparticles, biomass colloids, Individualized nanofibers, dynamic succinylation, imidazole chemistry

Table of Contents

1. Physical properties before and after functionalization S3
2. Materials S3
3. Preparation of cellulose nanofibrils (CNFs) S3
4. Chemical characterization of materials S6
5. Rheology, molar mass determination and structural characterization S9
6. Preparation of hydrogels, aerogels, and films S12
7. Mechanical characterization of nanopapers and hydrogels S13
8. Supporting References S15

1. Physical properties of the cellulose before and after functionalization

Table S1. Physical properties of the native cellulose fibers (reference), succinylated C6SA-cellulose and *nat*-cellulose (obtained upon ester hydrolysis of C6SA-cellulose).

	Reference	C6SA-Cellulose	*nat*-CNF$
Weight-averaged molar mass, M_w (kg/mol)	321	421	355
Z-averaged molar mass, M_z (kg/mol)	999	1641	1027
Weight-averaged degree of polymerization (DP)	1982	2203	2189
Crystallite size (nm)*	4.1	4.1	-
Crystallinity (%)*	55.6%	55.4%	-
Hemicellulose content (%)*	2.3%	1.4%	-
Degree of substitution (DS) (mmol/mmol)	-	0.25,# 0.24§	-
DS (C6OH) (mmol/mmol)	-	0.22§	-
Accessible primary hydroxyl groups (mmol/mmol)&	0.21	0.22	-

*Calculated from the deconvoluted solid-state NMR spectra, shown in **Figure 2E**. DS was measured by conductometric titration# and solution NMR§.& Accessible primary hydroxyl groups per cellulose monomer unit was calculated from the crystallite size, according to Okita *et al.*[1] $*nat*-CNF corresponds to the hydrolyzed C6SA-Cellulose obtained upon saponification.

2. Materials

Cellulose fibers of high purity were provided as never-dried bleached beech sulfite dissolving pulp (50 wt% solid content) by Lenzing AG (Lenzing, Austria) and used in the production of the succinylated pulp sample (C6SA-Cellulose). All chemicals were purchased from Sigma-Aldrich (Merck Life Science OY, Finland) at minimum purity of 99% and were used as received. Deionized (DI) water was used in all experiments and in the preparation of aqueous solutions and mixtures.

3. Preparation of cellulose nanofibrils (CNFs)

Optimization of reaction conditions

The reaction optimization was planned and analyzed with the program Design-Expert Version 11 from Stat-Ease, Inc (Minneapolis, Minnesota, USA). Screening tests were performed beforehand to identify the model limits for the optimization: amount of imidazole (1.0-1.5 equivalents (eq.) based on glucose monomer unit) and reaction time (0.25 h – 6.25 h). The optimization was performed with a randomized, quadratic response surface type model and a set of experimental conditions was predicted (**Table S2**). As response values, IR analysis was performed and the carbonyl band at 1730 cm^{-1} was selected to predict the amount of introduced succinate groups at the respective reaction conditions. The influence of Factor **A**, the amount of imidazole (molar equivalents based on glucose monomer unit) and Factor **B**, reaction time, in reactions with 1.0 eq. of succinic anhydride, acetone/water as solvent and a temperature of 40 °C is shown in **Figure S1**.

Table S2. Predicted reaction conditions and conducted runs for the optimization of the cellulose succinylation at a reaction temperature of 40 °C and 1.0 eq. (molar equivalent based on glucose monomer unit) of succinic anhydride. Factors A and B are varied in between the model limits. The intensity of the IR carbonyl band at 1730 cm^{-1} was used as response value.

Run	A: Imidazole (Eq.)*	B: Reaction time (h)	Response: IR carbonyl (a.u.)
1	1.3	0.25	0.12
2	1.5	3.25	0.127
3	1.1	6.25	0.133
4	1.3	6.25	0.132
5	1.1	3.25	0.121
6	1.5	0.25	0.115
7	1.5	6.25	0.136
8	1.3	3.25	0.12
9	1.1	0.25	0.117
10	1.3	3.25	0.121
11	1.3	3.25	0.128
12	1.3	3.25	0.119
13	1.3	3.25	0.12
14	1.3	3.25	0.123
15	1.3	3.25	0.122
16	1.5	6.25	0.133
17	1.1	3.25	0.123
18	1.3	0.25	0.112
19	1.1	6.25	0.129
20	1.5	0.25	0.117
21	1.5	6.25	0.136
22	1.1	0.25	0.113

*Equivalents based on glucose monomer unit.

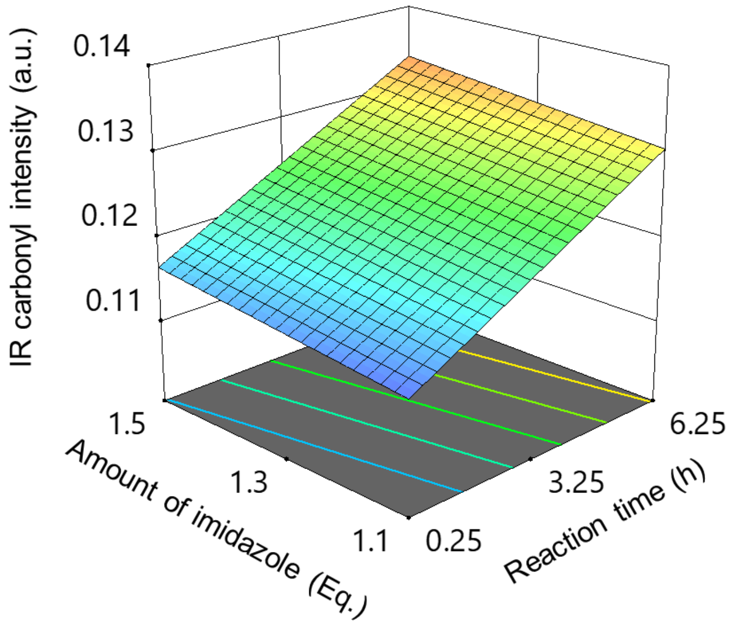

Figure S1. The influence of Factor **A**, the amount of imidazole (molar equivalents based on glucose monomer unit) and Factor **B**, reaction time, on the succinylation using 1.0 eq. of succinic anhydride, acetone/water as solvent and a temperature of 40 °C. The model equation from the optimization is $IR\ carbonyl\ intensity = 0.103667 + 0.008077\boldsymbol{A} + 0.002872\boldsymbol{B}$. The infrared (IR) carbonyl intensity was used as response corresponding to the final DS (intensity of 0.134 approximates a DS of 0.25). The equation can be used to finetune the DS of the C6SA-cellulose.

Preparation of regioselectively succinylated cellulose nanofibrils (CNFs)

Never-dried cellulose fibers (20.0 g wet mass, 10 g dry mass, 61.7 mmol, 50 wt% solid content) was transferred into a flask. In a separate container, 30.8 mL of a 3 M solution of imidazole (6.30 g, 92.5 mmol, 1.5 molar equivalents) in acetone were stirred with 61.7 ml of a 1 M succinic anhydride solution (6.17 g, 61.7 mmol, 1.0 molar equivalents) in acetone for 10 min. Afterwards this mixture was added to the cellulose fibers, and mixed by stirring with a glass rod for 1 min. The container was closed and heated in an oven at 40 °C for 6.25 h. The reaction was stopped through addition of a saturated aqueous solution of $NaHCO_3$ and 30 min equilibration. To remove the unreacted SA and the imidazole from the cellulose, the pulp was washed by filtration with water. The cellulose fibers were suspended in water at 0.25 wt% solid content with a blender. C6SA-cellulose suspension was fibrillated in a high-pressure homogenizer, Gaulin APV-1000 from AxFlow GesmbH (Premstätten, Austria). The homogenization of the fibers was done in 5 passes at a pressure of approx. 800 bar to yield a highly viscous and transparent dispersion of C6SA-CNF, which was stored at 8 °C.

Preparation of TEMPO-oxidized CNF (TO-CNF)

TO-CNF were prepared according to a procedure from the literature.[2] Never-dried cellulose (20 g, 10 g dry mass) was suspended in 0.05 M sodium phosphate buffer (900 mL, pH 6.8), TEMPO catalyst (0.016 g, 0.1 mmol) and sodium chlorite (80%, 1.13 g, 10 mmol) were added under stirring. A 2 M sodium hypochlorite solution (5 mL, 10 mmol) was diluted to 0.1 M in 0.05 M sodium phosphate buffer and was added to the reaction mixture. The flask was immediately stoppered, and the suspension was stirred at 500 rpm and 60 °C for 48 h. After cooling the suspension to room temperature, the TEMPO-oxidized cellulose was thoroughly washed with water by filtration. The fibers were dispersed at a solid content of 0.25 wt% and the pH was adjusted to 8 with an aqueous NaOH solution to deprotonate the carboxyl groups. Finally, the fibrillation was conducted using the same conditions as in the case of C6SA-CNF.

4. Chemical characterization of materials

Conductometric titration was used to determine the carboxylate content and the degree of substitution. For the titration 10.9 mg of freeze-dried C6SA-CNF was dispersed in 30 ml of water, 4 ml of a 0.01 M NaOH solution was added, and the mixture was stirred until the conductivity value was constant. The titration was performed using a Metrohm 856 Conductivity Module using a Mettler Toledo Seven Easy Conductivity Electrode (Greifensee, Switzerland). During the titration, a total of 8 ml of 0.01 M HCl was added in 0.05 ml steps. The plateau in the titration curve (**Figure S2**) was used to calculate the carboxylate content and the degree of substitution.

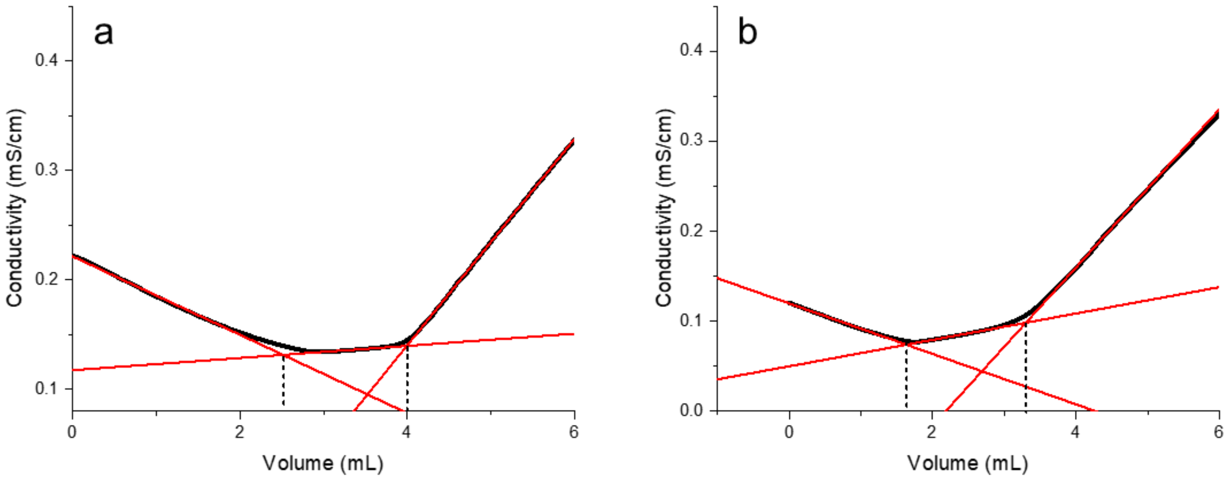

Figure **S2**: Conductivity titration curves of freeze-dried C6SA-CNF with 10 mM HCl. The degree of substitution (0.25) and carboxylate content (1.3 mmol/g) were determined from the plateau of the curves. The measurement was conducted in duplicate (a and b).

Infrared spectroscopy (IR). The IR measurements were performed on a PerkinElmer FT-IR Spectrometer Frontier (Waltham, Massachusetts, USA). Spectra were recorded from 4000 to 650 cm^{-1} with a resolution of 4 cm^{-1}. Advanced baseline correction (adaptive, coarseness 20 and offset 0) and normalization to highest peak was performed with the software Spectragryph 1.2.11.

Solution-state nuclear magnetic resonance (NMR) spectroscopy. To prepare the samples for NMR analysis, typically 50 mg of dried cellulosic material was added to a sealable sample vial and made up to 1 g by addition of stock [P_{4444}][OAc]:DMSO-d_6 (20:80 wt%) electrolyte solution.[3,4] The samples were magnetically stirred at room temperature until they went visually clear, which typically took ~1 hour. If the samples did not go clear during that period, the temperature was increased to 65 °C and when dissolved – transferred while hot into Wilmad 5 mm high-throughput tubes. All NMR runs were recorded on a Bruker AVANCE NEO 600 MHz spectrometer equipped with a 5-mm SmartProbe™ set to 65 °C. The diffusion-edited ^{1}H experiment used a 1D bipolar-pulse pair with stimulated echo (BPPSTE)[5] diffusion-ordered spectroscopy (DOSY) pulse sequence (Bruker pulse program ‘ledbpgp2s1d’), with 3 s relaxation delay (d1), 0.5 s acquisition time (aq), 16 dummy scans (ds), 128 transient scans (ns), a sweep-width (sw) of 20 ppm with the transmitter offset on 6.1 ppm (o1p), diffusion time (d20) of 200 ms, gradient recovery delay (d16) of 0.2 ms, eddy current delay (d21) of 5 ms, diffusion gradient pulse duration (p30) of 2.5 ms, and z-gradient strength (gpz6) of 90% at ^{3} 50 G/cm (probe z-gradient strength). These conditions are specific to the NMR apparatus above and may need reoptimization for other systems. The diffusion-edited ^{1}H spectra for the sample of C6SA-cellulose are shown below (Figure S3a, b; upper trace of 2D spectra). The degree of substitution was determined from peak fitting of the ^{1}H NMR C6SA-cellulose spectrum relating the protons from the glucose monomer unit to the protons of the succinyl group, respectively. The HSQC experiments used a multiplicity-edited phase-sensitive HSQC sequence with echo/antiecho-TPPI gradient selection (Bruker pulse program ‘hsqcedetgp’).[6] The parameters were as follows: spectral widths were 13 ppm and 165 ppm, with transmitter offsets (o1p) of 6.18 and 75 ppm, for ^{1}H and ^{13}C dimensions, respectively. The time-domain size (td1) in the indirectly detected ^{13}C-dimension (f1) was 512, corresponding to 256 t1-increments for the real spectrum. There were 16 dummy scans and 64 scans, an acquisition time of 0.065 s for f2 and a relaxation delay of 1.5 s. Sine squared (90°) window functions were used in f1 and f2. HSQC spectrum of the succinylated C6SA-cellulose is shown in Figure S3. The regioselectivity of the succinylation was determined by peak fitting of the ^{1}H NMR spectrum, according to a previously published work.[7]

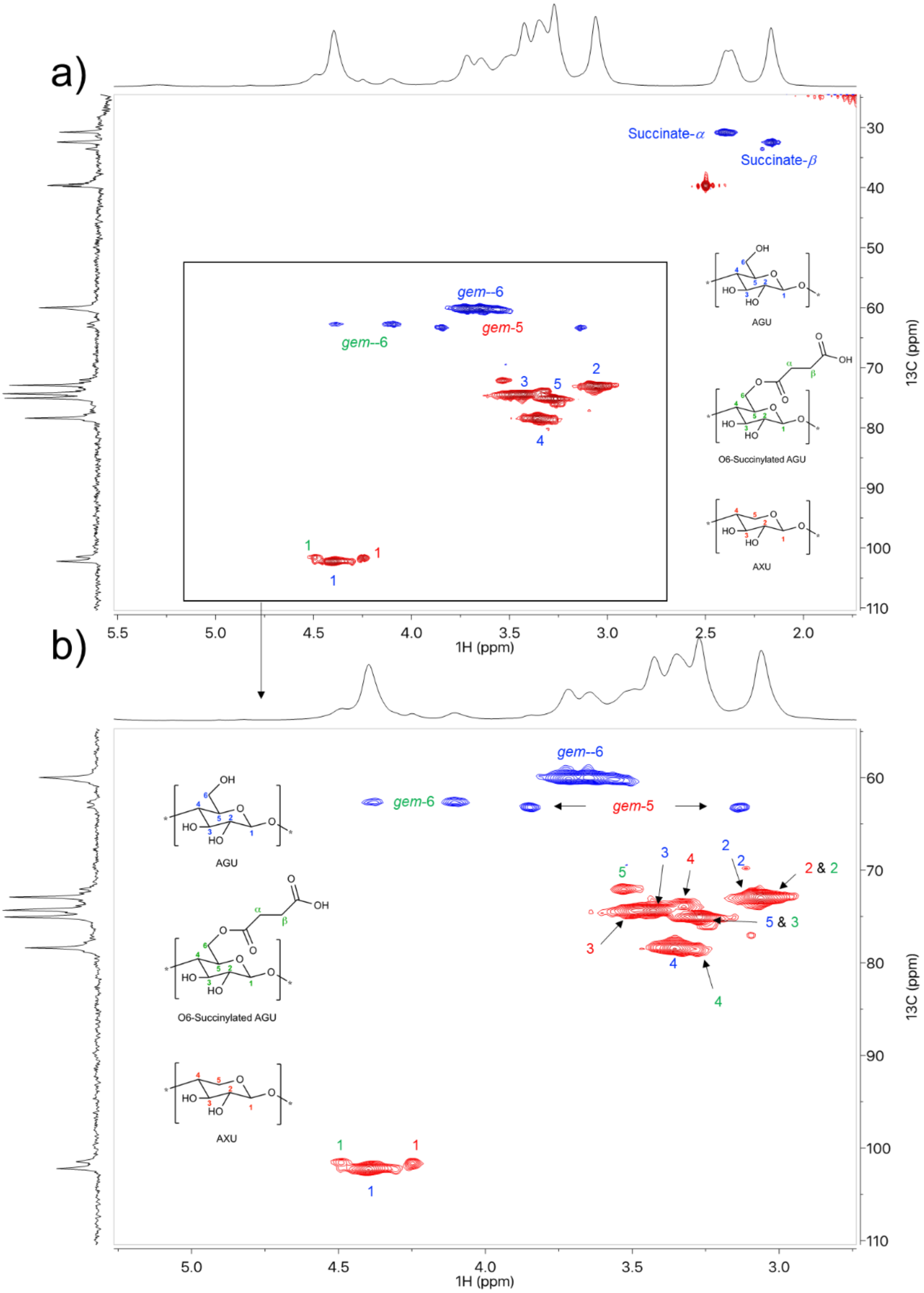

Figure **S3**: Multiplicity-edited heteronuclear single-quantum correlation (HSQC) 2D NMR spectrum (a) and zoom (b), including the peak assignments of native cellulose (nanofibril core, AGU), succinylated cellulose (nanofibril surface, C6-succinylated AGU) and hemicellulose traces (xylan, AXU).

Solid-state NMR spectroscopy. Solid state NMR experiments were measured on a Bruker Avance III HD 400 spectrometer (resonance frequency of ^{1}H of 400.13 MHz, and ^{13}C of 100.61 MHz, respectively), equipped with a 4 mm dual broadband CP-MAS probe. ^{13}C spectra were obtained by using the TOSS (total sideband suppression) sequence at ambient temperature with a spinning rate of 5000 Hz. The NMR experiment was conducted with a cross-polarization (CP) contact time of 2 ms, a recycle delay of 2 s, a SPINAL-64 ^{1}H decoupling and an acquisition time of 49 ms. The spectral width was set to 250 ppm. Chemical shifts were referenced externally against the carbonyl signal of glycine at $\delta = 176.03$ ppm. The acquired FIDs were apodised with an exponential function (lb = 11 Hz) prior to Fourier transformation. All materials for solid-state NMR were soaked in water as described by Zuckerstätter *et al.* before measurement.[8] For standard data processing the software Bruker TopSpin 3.5 was used. The crystallinity were determined by deconvolution of the crystalline and amorphous part of C4.[9] This method was used to estimate the effect of reaction conditions on the crystallinity of the samples. Peak fitting of native cellulose solid-state NMR spectrum was done according to Wickholm *et al.*[9] using the program Dmfit[10] and the elementary fibril diameter was calculated based on the work of Newman[11] using the average of the lateral cellulose chain spacings calculated from the 24 chain model (**Figure 2E** and **Figure S5**).[12] The resulting data from the fittings was used further to calculate as well the amount of hemicellulose in the sample and the crystallinity index in **Figure 2E**.

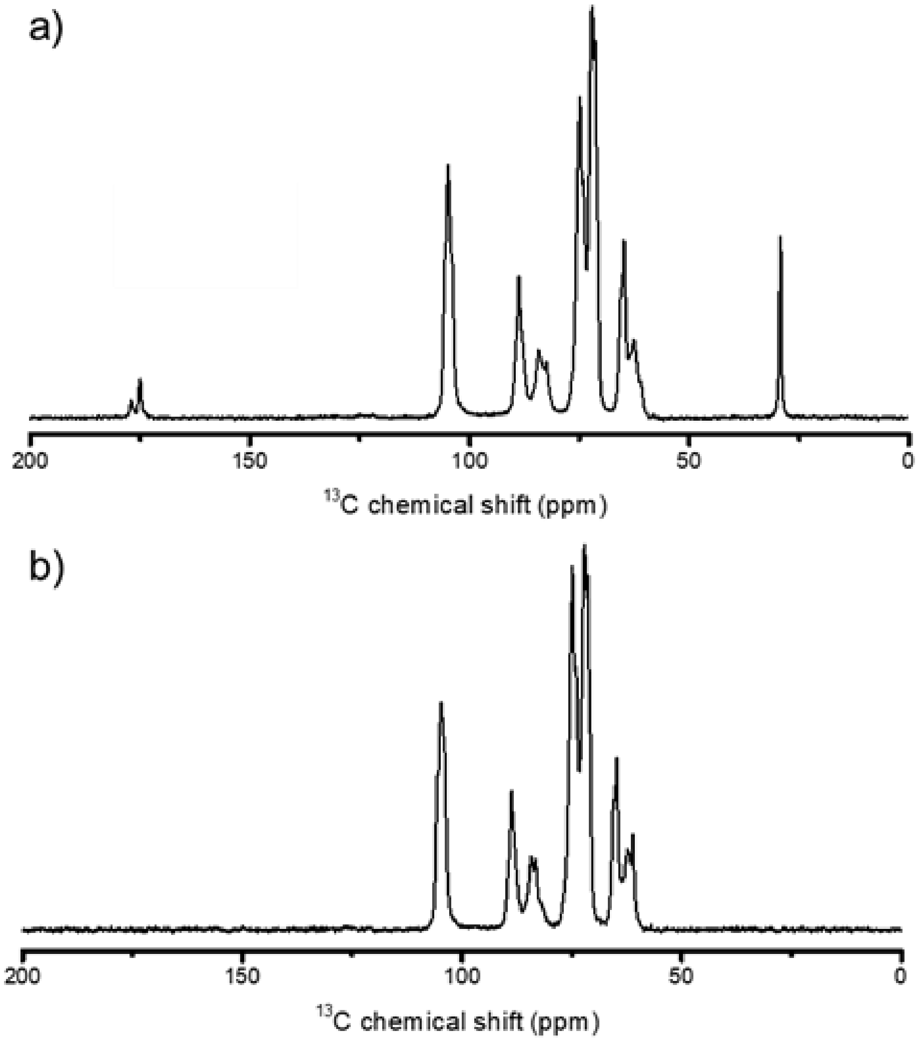

Figure S4: Solid-state ^{13}C NMR spectrum of C6SA-cellulose (a) and the native cellulose fibers (reference, b).

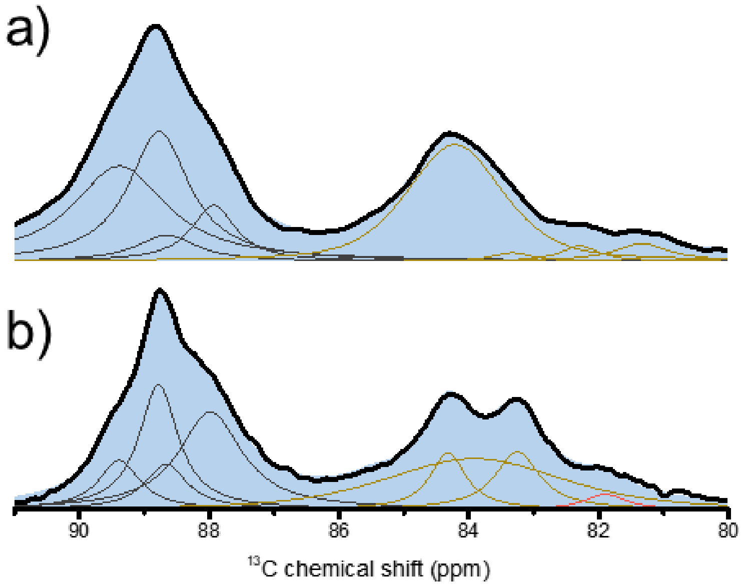

Figure S5: Deconvoluted C4 peak of the solid-state NMR spectrum of TEMPO-oxidized cellulose (a) in comparison to the native starting material (b).

5. Rheology, molar mass determination and structural characterization

Rheology. The rheology of the CNF suspensions was measured on an MCR 302 rheometer (Anton Paar, Austria), equipped with a plate-plate measurement system PP25 (Anton Paar, 25 mm diameter) at 20 °C. Viscosity measurements were conducted from 0.1–100 s^{-1}. Frequency sweep was measured from 100 to 0.5 rad/s at a strain amplitude of 0.1% (within linear viscoelastic region).

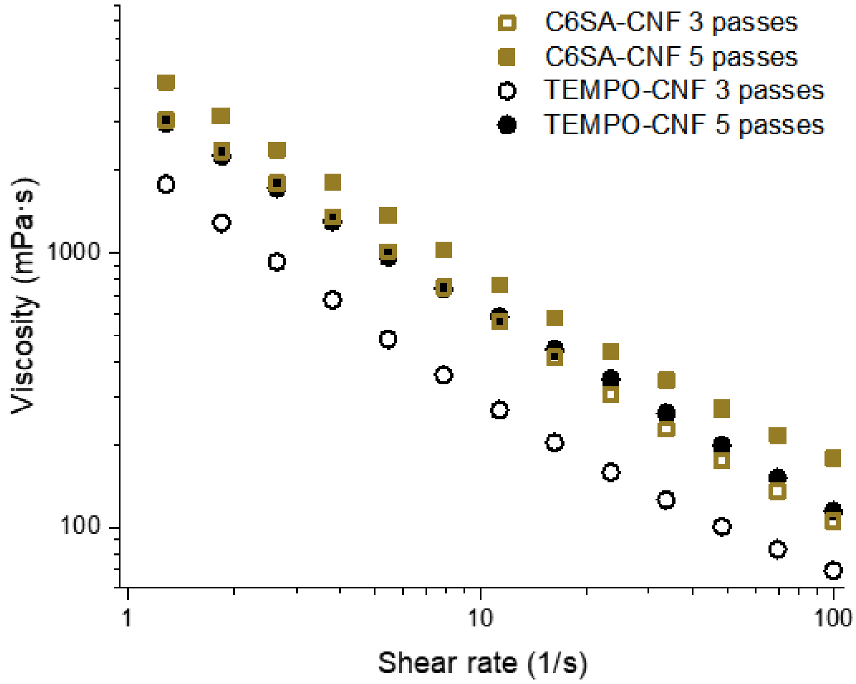

Figure S6: Influence of the number of passes through a high-pressure homogenizer on the viscosity of C6SA-CNF in comparison to TEMPO-CNF. The solid content of all samples was 0.25 wt%.

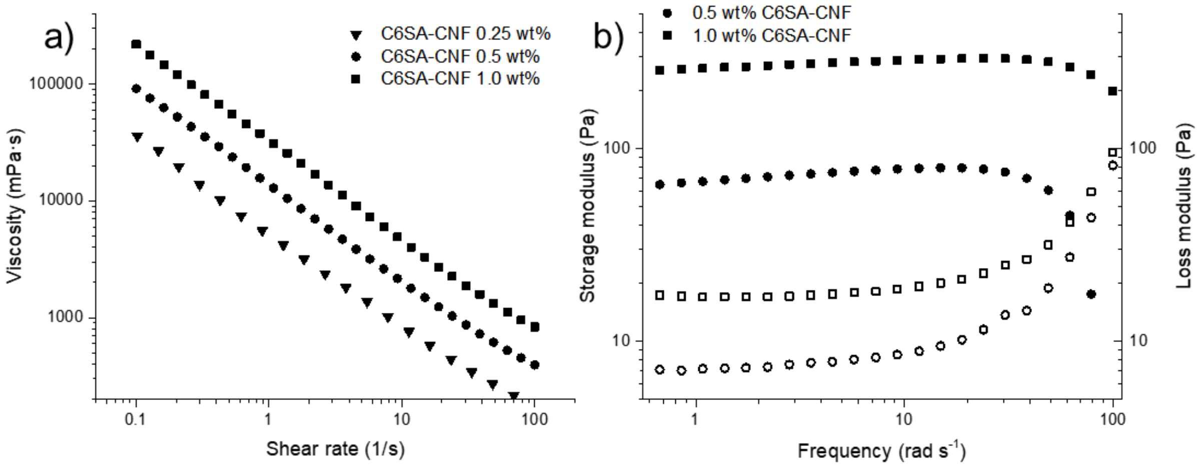

Figure S7: Rheological profiles of C6SA-CNF dispersions: Viscosity *vs*. shear rate (a) and, shear moduli *vs*. frequency (b).

Gel permeation chromatography (GPC). For GPC analysis, approximately 15 mg (dry equivalent) of never-dried pulp samples were dispersed in 250 mL of water and treated for approximately 20 s in a blender. One succinylated sample was treated with 4 mL of an aqueous NaOH solution (0.1 M) for 24 h. Throughout this treatment the sample was kept on a laboratory shaker. Then, the sample was washed by filtration with water and ethanol. The other samples were filtered and washed with ethanol. Each sample was transferred in a 4 mL vial, 4 mL of *N*,*N*-dimethylacetamide (DMAc) was added and the suspension was shaken overnight. Afterwards, the excess of DMAc was removed by filtration and 2 mL of DMAc/LiCl (9%, w/v) were added. The mixture was shaken at room temperature until complete dissolution of the cellulose sample. Finally, 0.3 mL of the sample were diluted with 0.9 mL DMAc and filtered through a 0.45 µm grid syringe filter. The GPC measurements were done with a multiple-angle laser light scattering (MALLS) detector with an argon ion laser (λ = 488 nm) (Wyatt Dawn DSP, Wyatt Inc. Santa Barbara, USA) and a refractive index (RI) detector (Shodex RI-71, Showa Denko K.K., Japan). Of every sample 100 µL were injected with an Agilent HP series 1100 autosampler (Agilent, Waldbronn, Germany). A Bio-Inert 1260 Infinity II (Agilent, Waldbronn, Germany) was used, with four serial GPC columns (Agilent PLgel Mixed ALS, 20 µm, 300 mm x 7.5 mm). The eluent was DMAc/LiCl (0.9%, w/v) at a flow rate of 1 mL/min and the run time was 45 min. The data was evaluated with Astra 4.7 and GRAMS/AI 7.0 software.

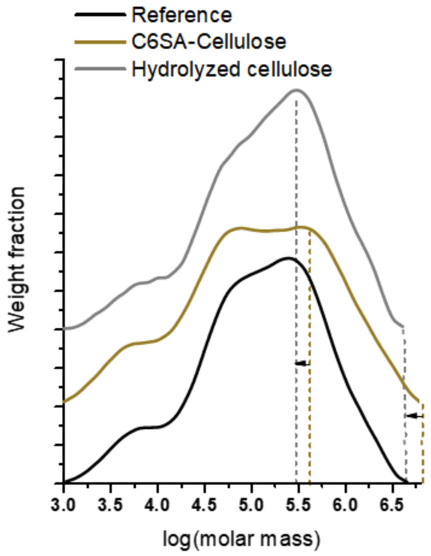

Figure S8: Molar mass distributions of reference sample, succinylated cellulose (C6SA-cellulose) and pristine sample, after alkaline hydrolysis (hydrolyzed cellulose, *nat*-cellulose). The shift of the chromatogram to lower mass values (indicated with black arrow) from C6SA-cellulose to hydrolyzed cellulose is another indication of the successful removal of the succinate group.

Atomic Force Microscopy (AFM). AFM micrographs were obtained on a Cypher AFM using a Herzian TS-150 active vibration table and an ARC2 SPM controller. Tapping mode was used at 1 Hz using Tap-300-G cantilevers (nominal radius of curvature 8 nm). The samples were prepared by first adsorbing poly(L-lysine) at pH 7 on freshly cleaved mica followed by thoroughly rinsing with Milli-Q water. C6SA-CNF at 0.04 wt% was prepared by dilution in Milli-Q water and dispersion with a tip sonicator (Digital Sonifier Model 450, Barbson Ultrasonics Corp.) for 2 min at 25% amplitude. A sessile drop of 50 µL of C6SA-CNF suspension at 0.04 wt% was spread over 1 cm^2. After 10-30 s, the samples were thoroughly rinsed with Milli-Q water and imaged in water without intermediate drying. The micrograph shown in Fig. 3C was shaded with an 8 degree azimuthal and polar angle to highlight the contour of the fibrils.

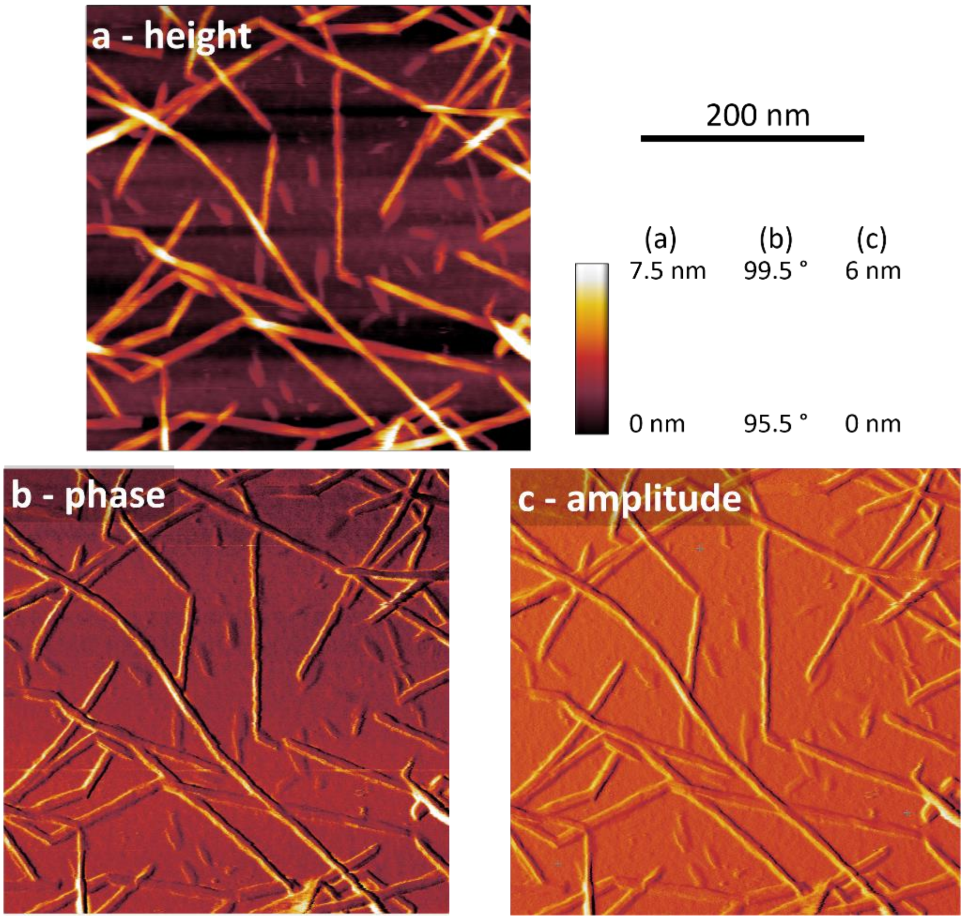

Figure S9: Height, phase, and amplitude images of the AFM micrograph in Figure 3A (Sample C6SA-CNF).

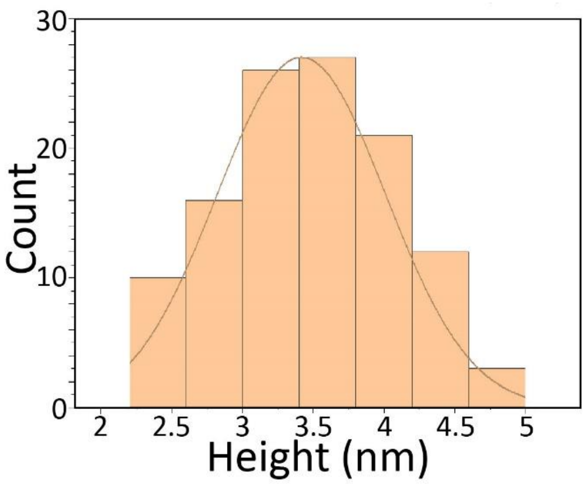

Figure S10: Distribution of height from a C6SA-CNF sample.

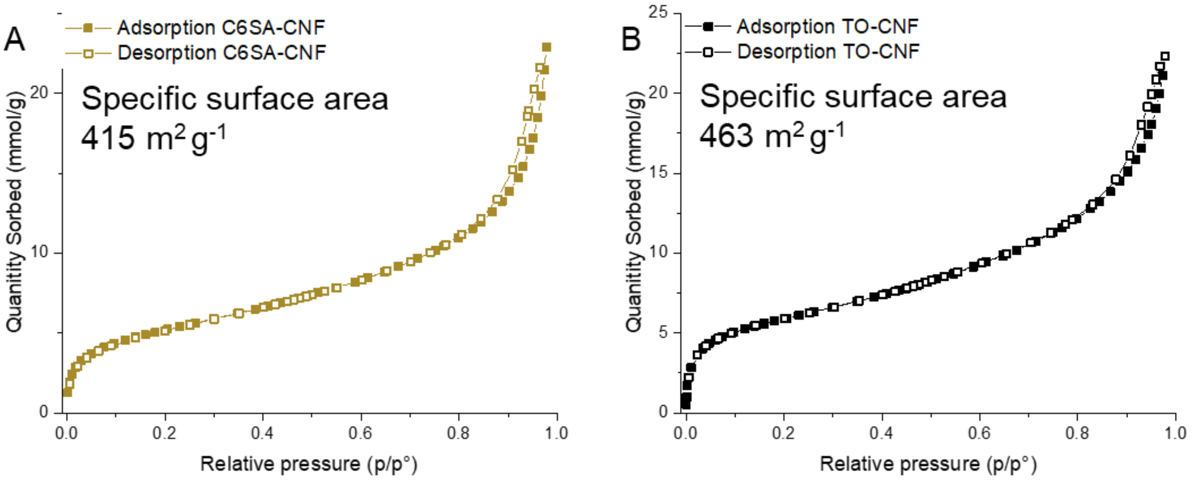

Figure S11: Nitrogen sorption curves (adsorption: filled symbols, and desorption: empty symbols) of C6SA-CNF aerogel (A) and TEMPO-oxidized CNF aerogel (TO-CNF, B). C6SA-CNF and TEMPO-oxidized CNF aerogels were prepared under the same conditions. In the literature, significantly higher surface areas of TEMPO-oxidized CNF (500 - 600 $m^2 g^{-1}$)[13] have been reported, which demonstrates the large influence of processing conditions, e.g., solid content of CNF dispersions prior to drying. Hence, the specific surface areas of C6SA-CNF and TEMPO-oxidized CNF aerogels are in a comparable range and can be significantly elevated through further process optimization.

Scanning Electron Microscopy. SEM micrographs of aerogels were obtained using a field emission scanning electron microscope (FE-SEM, Zeiss SigmaVP, Germany) operating at 1.6 kV and at a working distance of ca. 5 mm. The samples were sputter-coated with a 5 nm layer of platinum/palladium alloy prior to imaging.

6. Preparation of hydrogels, aerogels, and films

Preparation of C6SA-CNF hydrogels and aerogels. C6SA-CNF hydrogels (approx. 0.7 wt%) were either prepared by acid treatment in 0.01 M HCl or alkali treatment in 0.1 M NaOH for approx. 12 h. Both treatments induced a crosslinking, either by protonation of the sodium carboxylate groups or through saponification (removal of succinate esters). The prepared hydrogels were then solvent-exchanged to acetone to obtain their respective aerogels: the first solvent-exchange was conducted with acetone:water (1:1, v:v) for at least 12 h, then the gels were equilibrated in pure acetone (3 times, each for at least 12 h). In all cases, the volume of the solvent used for exchange was approx. 10 times the volume of the gel. The solvogels were then dried in a Leica EM CPD300 critical point dryer and the acetone was replaced with supercritical CO_2 over 25 cycles at 35 °C and 75 bar. Nitrogen adsorption and desorption isotherms were recorded on a TriStar II from Micromeritics at 77 K after degassing the cellulose powders under vacuum at 80 °C for 12 h (VacPrep 061 degasser, Micromeritics). The specific surface area was determined from 16 data points corresponding to the linear range (P/P_0 = 0.05-0.3)[14] of the adsorption branch using the Brunauer–Emmett–Teller (BET) method.[15] Mesoporosity of the samples was evaluated by the BJH (Barrett–Joyner–Halenda) method[16] based on the modified Kelvin equation, using the Broekhoff-De Boer model.[17] Nitrogen sorption measurements of C6SA-CNF aerogels were performed in duplicate and compared to the TEMPO-CNF aerogel, which was prepared analogously (**Figure S11**).

Preparation of nanopapers. The suspensions of TO-CNF and C6SA-CNF at 0.2 wt% were diluted to a 0.1% solid content by the addition of water and mixed in a magnetic stirrer (12 hours, 700 rpm). Before filtration, each sample was sonicated three times (30% amplitude, 5 min) using a digital sonicator (Branson Ultrasonics Sonifier™ S-250D Digital Ultrasonic Cell Disruptor/Homogenizer). Filtration was carried using a pressurized air system (4 bar, 16 h) in a self-made filtration device (**Figure S14**) consisting of a tripod chamber (inner diameter of 12 cm, height 8.5 cm). A polyvinylidene fluoride (PVDF) membrane filter (Durapore®, 142 mm diameter, 0.22 µm pore size, REF GVWP14250, Merck Millipore©), and a Schleicher & Schüll Rundfilter (150 mm diameter, Whatman™, Ref. No. 300212) were used as fitlers. The obtained hydrogel cakes after filtration were inserted in between a series of covering layers. These covering layers were composed of a PVDF membrane filter (the same as used in the filtration step), one layer of SEFAR NITEX® fabric (code: 03-1/1), four layers of regular bond paper, three layers of strawboard, and an aluminum plate. These covered fitler cakes were hot-pressed in a Carver Laboratory Press 18200-213 (Freds Carver Inc. Hydraulic equipment, NJ, USA) at 1500 lb and 80 °C for 50 min. The prepared circular nanopapers had a diameter of 120 mm and thickness of approx. 50 µm. The layer configuration and other characteristics of the filtration system are shown in **Figure S14**.

7. Mechanical characterization of nanopapers and hydrogels

The mechanical properties of the nanopapers and hydrogels were evaluated using a Universal Tensile Tester Instron 4204 with 1 kN load cell at a test speed of 20 mm/min. The specimens for testing were prepared according to the ASTM D638-03 standard. The dry samples (nanopapers) were stored before the test in a conditioned room at 50 % relative humidity and 23 °C for 48 h and tested without any additional treatment. In case of the water-swollen films (hydrogels), nanopapers were swollen and equilibrated in the respective solutions: Water, 0.01 M HCl (acidic treatment), or 0.1 M NaOH (alkali treatment). After acid and alkali treatment, the hydrogels were washed with water to remove salts. Dry measurements of the base-treated sample was carried out after hot-pressing (80 °C at 1500 lb for 50 min).

The sample tests were carried out on pre-cut strips (5.3 mm x 30 mm) fixed to the Instron clamps, using sandpaper glued to the Instron clamps with UHU® removable adhesive putty. The thickness of the samples was measured using a micrometer (Mitutoyo Quickmike Series 293-IP-54, resolution 1 µm, EU), these results were also used to evaluate the swelling of CNFs (**Figure S12**). The mechanical test was repeated ten times for each sample. After eliminating the outliers (e.g., wrongly placed samples, or damaged films from the sample preparation), at least six replicas of each sample were used for statistical analysis. Density and apparent porosity were obtained from gravimetric measurements, see **Table S3**. The apparent porosity was calculated by Equation 1 using a reference density of cellulose I (ρ_c = 1.55 g·cm^{-3})[18].

$$Porosity = \frac{\rho_a}{\rho_c} \times 100 \qquad (1)$$

where ρ_a is the apparent density (g·cm^{-3}), and ρ_c is the reference cellulose density.

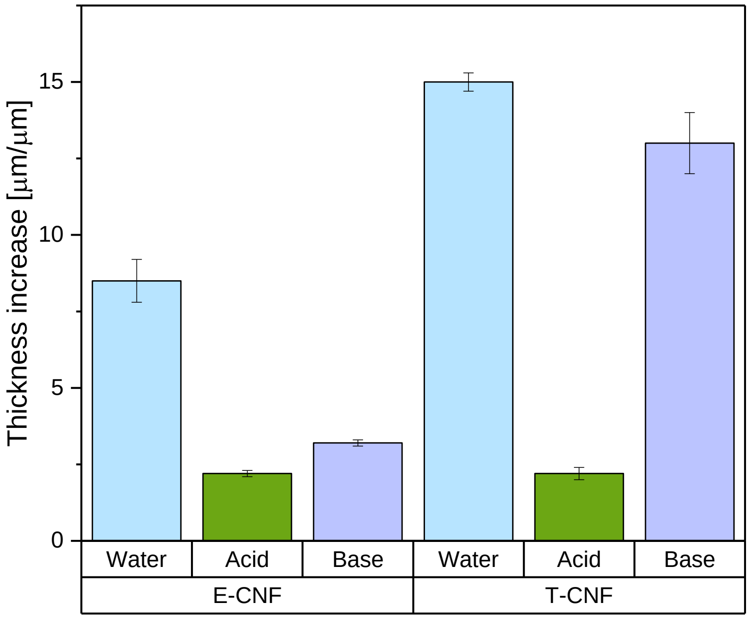

Figure S12: Swelling of C6SA-CNF and TO-CNF hydrogels (water-swollen nanopapers) and influence on thickness upon different treatments. All measurements were conducted after equilibration in water, remaining acid or base from the treatment was washed out beforehand.

Table S3. Densities and relative porosities of the produced dry films (non-treated vs. base-treated).

Sample	Conditions	Apparent density (g cm^{-3})	Relative porosity
C6SA-CNF	Native	1.54 ± 0.01	0.9 ± 0.4
nat-CNF	Base treatment	1.31 ± 0.04	15.5 ± 2.6

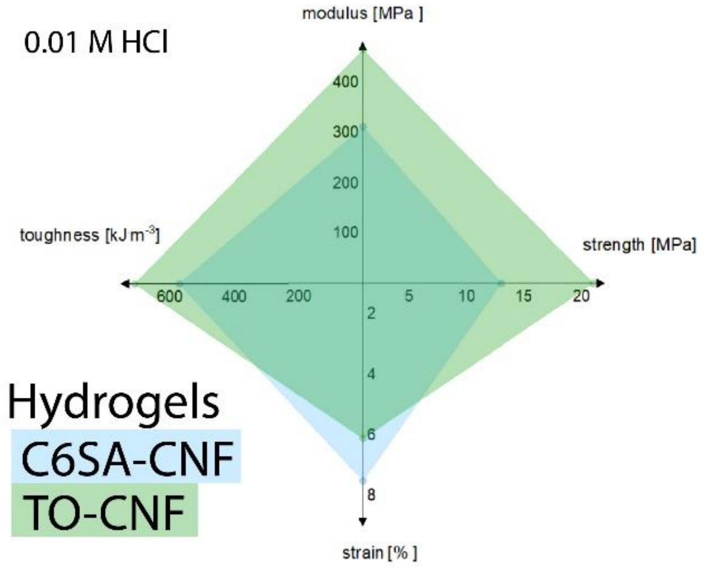

Figure S13: Tensile properties of hydrogels (water-swollen nanopapers) of C6SA-CNF and TO-CNF in their protonated state.

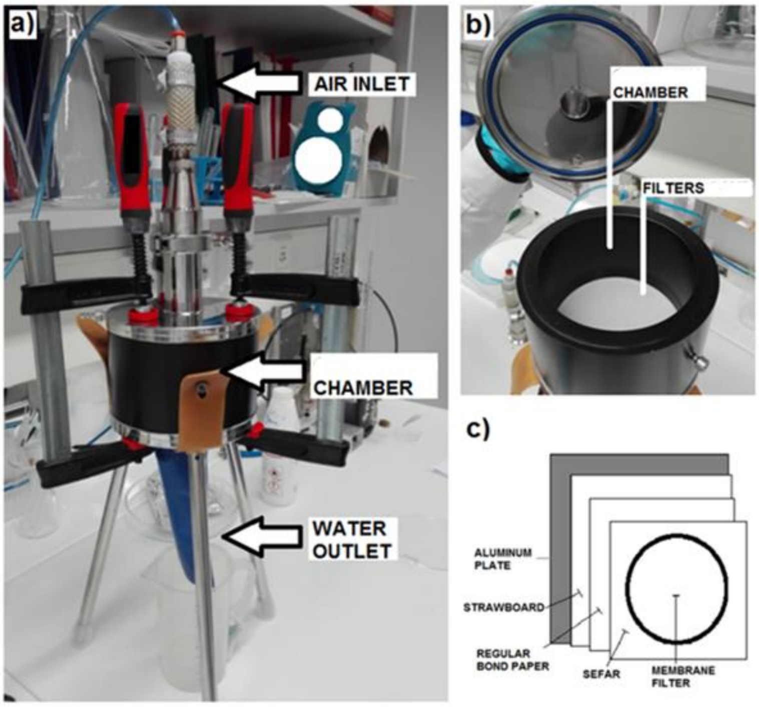

Figure S14. Equipment for preparation of nanopapers: a) Vacuum filtration unit composed of a b) chamber and filters. C) The samples were hot pressed in between a series of covering layers at each side of the hydrogel cake: A PVDF membrane filter, one layer of SEFAR fabric, four layers of regular bond paper, three layers of strawboard, and an aluminum plate.

8. Supporting References

(1) Okita, Y.; Saito, T.; Isogai, A. Entire Surface Oxidation of Various Cellulose Microfibrils by TEMPO-Mediated Oxidation. *Biomacromolecules* **2010**, *11* (6), 1696–1700. https://doi.org/10.1021/bm100214b.

(2) Saito, T.; Hirota, M.; Tamura, N.; Kimura, S.; Fukuzumi, H.; Heux, L.; Isogai, A. Individualization of Nano-Sized Plant Cellulose Fibrils by Direct Surface Carboxylation Using TEMPO Catalyst under Neutral Conditions. *Biomacromolecules* **2009**, *10* (7), 1992–1996. https://doi.org/10.1021/bm900414t.

(3) Koso, T.; Rico del Cerro, D.; Heikkinen, S.; Nypelö, T.; Buffiere, J.; Perea-Buceta, J. E.; Potthast, A.; Rosenau, T.; Heikkinen, H.; Maaheimo, H.; Isogai, A.; Kilpeläinen, I.; King, A. W. T. 2D Assignment and Quantitative Analysis of Cellulose and Oxidized Celluloses Using Solution-State NMR Spectroscopy. *Cellulose* **2020**, *27* (14), 7929–7953. https://doi.org/10.1007/s10570-020-03317-0.

(4) King, A. W. T.; Mäkelä, V.; Kedzior, S. A.; Laaksonen, T.; Partl, G. J.; Heikkinen, S.; Koskela, H.; Heikkinen, H. A.; Holding, A. J.; Cranston, E. D.; Kilpeläinen, I. Liquid-State NMR Analysis of Nanocelluloses. *Biomacromolecules* **2018**, *19* (7), 2708–2720. https://doi.org/10.1021/acs.biomac.8b00295.

(5) Wu, D. H.; Chen, A. D.; Johnson, C. S. An Improved Diffusion-Ordered Spectroscopy Experiment Incorporating Bipolar-Gradient Pulses. *J. Magn. Reson. A* **1995**, *115* (2), 260–264. https://doi.org/10.1006/jmra.1995.1176.

(6) Willker, W.; Leibfritz, D.; Kerssebaum, R.; Bermel, W. Gradient Selection in Inverse Heteronuclear Correlation Spectroscopy. *Magn. Reson. Chem.* **1993**, *31* (3), 287–292. https://doi.org/10.1002/mrc.1260310315.

(7) Beaumont, M.; Jusner, P.; Gierlinger, N.; W. T. King, A.; Potthast, A.; Rojas, O. J.; Rosenau, T. Unique Reactivity of Nanoporous Cellulosic Materials Mediated by Surface-Confined Water. *Nat. Commun.* **2021**, *12*, 2513. https://doi.org/10.1038/s41467-021-22682-3.

(8) Zuckerstätter, G.; Terinte, N.; Sixta, H.; Schuster, K. C. Novel Insight into Cellulose Supramolecular Structure through 13C CP-MAS NMR Spectroscopy and Paramagnetic Relaxation Enhancement. *Carbohydr. Polym.* **2013**, *93* (1), 122–128. https://doi.org/10.1016/j.carbpol.2012.05.019.

(9) Wickholm, K.; Larsson, P. T.; Iversen, T. Assignment of Non-Crystalline Forms in Cellulose I by CP/MAS 13C NMR Spectroscopy. *Carbohydr. Res.* **1998**, *312* (3), 123–129. https://doi.org/10.1016/S0008-6215(98)00236-5.

(10) Massiot, D.; Fayon, F.; Capron, M.; King, I.; Le Calvé, S.; Alonso, B.; Durand, J.-O.; Bujoli, B.; Gan, Z.; Hoatson, G. Modelling One- and Two-Dimensional Solid-State NMR Spectra. *Magn. Reson. Chem.* **2002**, *40* (1), 70–76. https://doi.org/10.1002/mrc.984.

(11) Newman, R. H. Estimation of the Lateral Dimensions of Cellulose Crystallites Using 13C NMR Signal Strengths. *Solid State Nucl. Magn. Reson.* **1999**, *15* (1), 21–29. https://doi.org/10.1016/S0926-2040(99)00043-0.

(12) Oehme, D. P.; Downton, M. T.; Doblin, M. S.; Wagner, J.; Gidley, M. J.; Bacic, A. Unique Aspects of the Structure and Dynamics of Elementary Iβ Cellulose Microfibrils Revealed by Computational Simulations. *Plant Physiol.* **2015**, *168* (1), 3–17. https://doi.org/10.1104/pp.114.254664.

(13) Kobayashi, Y.; Saito, T.; Isogai, A. Aerogels with 3D Ordered Nanofiber Skeletons of Liquid-Crystalline Nanocellulose Derivatives as Tough and Transparent Insulators. *Angew. Chem. Int. Ed.* **2014**, 10394–10397. https://doi.org/10.1002/anie.201405123.

(14) ISO. Determination of the Specific Surface Area of Solids by Gas Adsorption - BET Method. *ISO Stand.* **2010**, *9277*.

(15) Brunauer, S.; Emmett, P. H.; Teller, E. Adsorption of Gases in Multimolecular Layers. *J. Am. Chem. Soc.* **1938**, *60* (2), 309–319. https://doi.org/10.1021/ja01269a023.

(16) Thommes, M.; Kaneko, K.; Neimark, A. V.; Olivier, J. P.; Rodriguez-Reinoso, F.; Rouquerol, J.; Sing, K. S. W. Physisorption of Gases, with Special Reference to the Evaluation of Surface Area and Pore Size Distribution (IUPAC Technical Report). *Pure Appl. Chem.* **2015**, *87* (9–10), 1051–1069. https://doi.org/10.1515/pac-2014-1117.

(17) Broekhoff, J. C. P.; de Boer, J. H. Studies on Pore Systems in Catalysts: XIII. Pore Distributions from the Desorption Branch of a Nitrogen Sorption Isotherm in the Case of Cylindrical Pores B. Applications. *J. Catal.* **1968**, *10* (4), 377–390. https://doi.org/10.1016/0021-9517(68)90153-X.

(18) Dufresne, A. Nanocellulose: A New Ageless Bionanomaterial. *Mater. Today* **2013**, *16* (6), 220–227. https://doi.org/10.1016/j.mattod.2013.06.004.
